# Highly Efficient Thermoresponsive Nanocomposite for Controlled Release Applications

**DOI:** 10.1038/srep28539

**Published:** 2016-06-23

**Authors:** Omar Yassine, Amir Zaher, Er Qiang Li, Ahmed Alfadhel, Jose E. Perez, Mincho Kavaldzhiev, Maria F. Contreras, Sigurdur T. Thoroddsen, Niveen M. Khashab, Jurgen Kosel

**Affiliations:** 1 Computer, Electrical and Mathematical Sciences & Engineering Division, King Abdullah University of Science and Technology (KAUST), Thuwal 23955-6900, Kingdom of Saudi Arabia; 2 School of Engineering, University of British Columbia, 3333 University Way, Kelowna, BC, V1V 1V7, Canada; 3 Physical Sciences and Engineering Division, King Abdullah University of Science and Technology, Thuwal 23955-6900, Kingdom of Saudi Arabia; 4 Biological and Environmental Sciences and Engineering Division, King Abdullah University of Science and Technology, Thuwal 23955-6900, Kingdom of Saudi Arabia; 5 Smart Hybrid Materials Laboratory, Advanced Membranes and Porous Materials Center, King Abdullah University of Science and Technology, Thuwal 23955-6900, Kingdom of Saudi Arabia

## Abstract

Highly efficient magnetic release from nanocomposite microparticles is shown, which are made of Poly (N-isopropylacrylamide) hydrogel with embedded iron nanowires. A simple microfluidic technique was adopted to fabricate the microparticles with a high control of the nanowire concentration and in a relatively short time compared to chemical synthesis methods. The thermoresponsive microparticles were used for the remotely triggered release of Rhodamine (B). With a magnetic field of only 1 mT and 20 kHz a drug release of 6.5% and 70% was achieved in the continuous and pulsatile modes, respectively. Those release values are similar to the ones commonly obtained using superparamagnetic beads but accomplished with a magnetic field of five orders of magnitude lower power. The high efficiency is a result of the high remanent magnetization of the nanowires, which produce a large torque when exposed to a magnetic field. This causes the nanowires to vibrate, resulting in friction losses and heating. For comparison, microparticles with superparamagnetic beads were also fabricated and tested; while those worked at 73 mT and 600 kHz, no release was observed at the low field conditions. Cytotoxicity assays showed similar and high cell viability for microparticles with nanowires and beads.

Poly (N-isopropylacrylamide) (PNIPAM) is a stimuli-responsive or “smart” microgel that swells by uptaking a solvent (e.g. water) below its transition temperature, called the lower critical solution temperature (LCST), and shrinks above this LCST by expelling the solvent^1^. These microgels can respond to stimuli such as temperature, pH, light and electric fields^2^,^3^,^4^,^5^,^6^. Due to their distinct properties, responsive microgels have been employed in various applications including sensing, catalysis, drug delivery, optical devices, cell attachment and culturing, radiotherapy and optics^7^,^8^,^9^,^10^,^11^,^12^.

The functionalities of PNIPAM can be further modified by embedding micro or nanoparticles that are responsive to different types of stimulations, including magnetic fields^5^,^13^,^14^,^15^,^16^,^17^,^18^. Compared to externally applied direct thermal stimulation that heats the entire area of operation, or integrated heaters that require complex fabrication steps, utilizing magnetic losses from embedded magnetic nanobeads (NBs) provides highly localized and remotely controlled PNIPAM triggering. While this approach has been successfully implemented for various applications, the drawback is primarily the low heating efficiency, i.e. the need for high magnetic fields (50 mT to 100 mT) and high frequencies (300 kHz to 1000 kHz)^19^,^20^. Heat production from superparamagnetic losses is generally proportional to the frequency of the magnetic field, which causes the magnetic hysteresis loop to widen, increasing the heat generation per magnetic field cycle^21^,^22^. Frequencies at which measurable heating occurs depend on the suspension medium type and the particle geometry^21^,^22^. The higher power requirements hinder further development of practical applications of such materials, because these high fields cannot be produced on-chip in the case of integrated devices, and require large water cooled coils and high-power sources in the case of applications like drug delivery in human patients.

In this work, we propose a new thermoresponsive nanocomposite material made of PNIPAM microparticles with embedded iron nanowires (NWs). Magnetic heating is based on friction rather than magnetic losses (Néel relaxation and Brownian relaxation in case of NBs)^23^, enabling heating of these nanowire composite (NWC) particles with only a fraction of the fields and frequencies required for nanobead composite (NBC) particles, made of PNIPAM with iron oxide NBs. The lower power requirement stems from the high remanent magnetization of iron NWs, which is a result of their single magnetic domain behavior^24^,^25^,^26^. It permits the generation of enough torque in the NWs at low fields to produce frictional losses with their surroundings, which dissipate as heat into the entire NWC particle. A model describing the angular changes of the NWs during the alternating magnetic field (AMF) induced vibration is used to calculate the magnetically applied torque and the total work input into the microparticle over time (see Supplementary Material). This work input is converted to heat generation, which is transferred from the NWs to its surroundings by friction. The losses found by this model match those found for magnetic losses in NBC particles (see Supplementary Material).

A simple yet efficient microfluidic technique for fabricating NBC particles, using a glass capillary microchip^27^, was adopted to fabricate NWC particles. This approach allows thermoresponsive NWC particles to be fabricated with a high control of the concentration and in relatively short time compared to chemical synthesis methods^20^. Weitz’s group was among the first to prove the efficiency and simplicity of this technology for embedding different kinds of micro and nanoparticles. This concept was then used for remotely triggered release applications.

For example, Li *et al*.^17^ embedded polypyrrole nanoparticles as photo-thermal transducers PNIPAM, which are triggered by near-infrared-light irradiation, providing a simple method for optically-controlled and localized delivery of neuro-active substances such as neurotransmitters. Similarly. Jadhav *et al*.^28^ used a microfluidic chip to fabricate a photo-stimulated valve which can be switched on in a time of around just one second and switched off in around six seconds. Luo *et al*.^29^,^30^ fabricated agarose/alginate double network hydrogels and multi-compartmental hydrogel particles with robust mechanical propreties. In addition, Jia *et al*.^31^ combined microfluidic and centrifugation-re-dispersion technologies to form core/shell photonic crystal microbeads by using an assembly made of PNIPAM and polystyrene nanoparticles for optical encoding applications. Seo *et al*.^32^ incorporated magnetic nanoparticles in hydrogel microparticles to fabricate flexible and location traceable organo-motor. Also, Wei *et al*.^33^ used this technology to fabricate multi-responsive microcapsules for adjustable and controlled release applications through combining magnetic, pH and thermoresponsive particles.

In this work we use for the first time the microfluidic fabrication approach as a simple and fast method to embed magnetic nanowires into thermoresponsive PNIPAM microparticles.

The NWC particles were used for the remotely triggered release of Rhodamine (B), (Rh (B)), which was employed as the model aqueous drug. A magnetic field of only 1 mT and 20 kHz was applied to achieve a drug release of approximately 6.5% and 70% in the continuous and pulsatile modes, respectively. Compared to the values typically reported in literature for NBC particles the drug release from NWC particles is achieved with a magnetic field that is about five orders of magnitude lower in power. This dramatic decrease in power requirement allows realizing applications and devices in a much more compact, efficient and cheaper way.

The concept of magnetically triggered drug release with NWC particles is presented in (Fig. 1). The particles are fabricated in a microfluidic device and are collected and washed prior to drug loading. The drug is released from the magneto-thermoresponsive PNIPAM particles by applying an AMF.

The radius of the fabricated NWC and NBC particles ranged from 20 to 200 μm. All results reported here were achieved with particles of 50 μm in radius, if not mentioned otherwise, a NW concentration of 2.54% v/v and a NB concentration of 2.54% v/v (NBC_(2.54)_) and 0.175% v/v (NBC_(0.175)_). While NBC_(2.54)_ was used for comparison with NWC with the same concertation values, NBC_(0.175)_ allows a comparison with NWC under the condition of similar heating power and timescales, when applying a high-power magnetic field of 73 mT and 600 kHz to the NBC particles and a low-power magnetic field of 1 mT and 20 kHz to the NWC particles.

## Results and Discussion

### Thermoresponsive behavior

**Figure 2:**
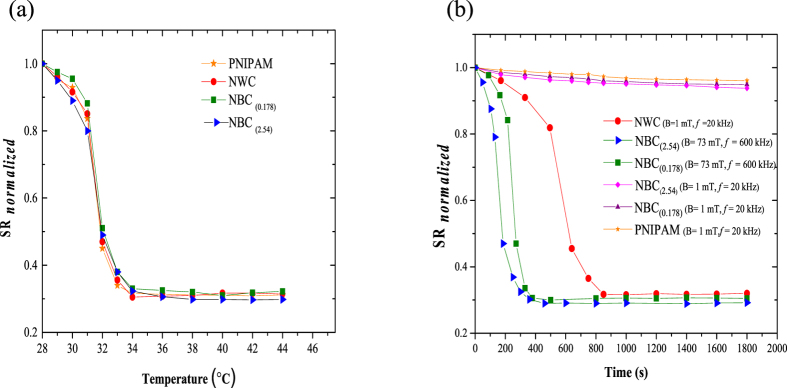
(**a**) Swelling/deswelling ratio (normalized) of pure PNIPAM particles and of magneto-thermoresponsive nanowire composite (NWC) particles (2.54% v/v) or nanobeads composite (NBC) particles with 0.175% v/v (NB _0.175_) and 2.54% v/v (NBC_2.54_). The experiments were performed by direct heating: the particles were immersed in a temperature controlled water bath with no magnetic field application. (**b**) Swelling/deswelling ratio (normalized) of particles as a response to an alternating magnetic field. The field parameters and concentrations of NBs are shown in brackets. PNIPAM and NBC particles do not respond to a low-power field as opposed to NWC particles, which deswell. NBC_(2.54)_ and NBC_(0.175)_ particles respond only to a high-power field.

In order to study the effect of embedding the NWs and NBs into PNIPAM, we compared the swelling-deswelling behavior of native PNIPAM particles to that of NWC and NBC particles. To this end, direct heating experiments with no magnetic field were performed with particles inside a water bath with a temperature from 28 °C to 42 °C. The normalized swelling ratio (SR_*Normalized*_) of the different particles as a function of the temperature is shown in (Fig. 2a). Nearly the same behavior is observed for PNIPAM, NWC and NBC particles at different concentrations. This implies that integrating magnetic NWs or NBs in the PNIPAM microgel, using the microfluidic fabrication method, leaves its thermoresponsive behavior unchanged. This is in contrast to results obtained with chemical synthesis methods, where the NBs are chemically linked to the gel network, causing a decrease in the sensitivity of PNIPAM to thermal stimuli^34^. It seems, by using the microfluidic approach the NBs are more physically linked to the gel network, hence maintaining efficient responsive behavior^27^. This property could be exploited to generate a variety of PNIPAM particles with embedded nanomaterials of different size or concentration, each optimized for specific applications.

From the SR plot in Fig. 2a, the LCST is estimated to be approximately 31 °C, which is in accordance with typical values reported in literature^1^.

### Magnetoresponsive behavior

The magnetoresponsive properties of the particles were studied by applying an alternating magnetic field (AMF) of either low power (1 mT, 20 kHz) or high power (73 mT, 600 kHz) and measuring the SR. As shown in Fig. 2b, PNIPAM particles do not respond to an AMF. NBC_(2.54)_ and NBC_(0.175)_ particles respond to the high power field in a similar way as to direct heating (Fig. 2a), whereby the particles with higher NB concentration shrink faster, due to higher heating power^19^. Neither of the NBC particles responds to the low power AMF. This is in contrast to the NWC particles, which show a slight decrease during the first six minutes, followed by a sharp decrease at around 550 seconds, which indicates the temperature reached the LCST value. Eventually, the SR saturates after about 850 seconds, when maximum shrinking is achieved. These observations can be explained by calculating the power densities (Supplementary material) for NBC_(2.54)_ and NWC particles exposed to the lower power field, which are 1.76 × 10^−6^ W/m^3^ and 4.84 × 10^5^ W/m^3^, respectively. Hence, NBC particles do not respond to the magnetic trigger even at 2.54% v/v iron oxide concentration. The magneto-responsive behavior of the NWC particles is also similar to the one obtained from direct thermal stimuli (Fig. 2a).

### Drug release

Loading the particles by physical diffusion with Rhodamine (B) (Rh(B)), used as the aqueous drug model, yielded a load efficiency *Load*_eff_ of 30%. The drug release was studied for a continuous mode, i.e. an AMF was continuously applied for a specific period of time, and for a pulsatile mode, i.e. the AMF was sequentially applied with a break period between the application pulses. The release experiments are conducted in this way to highlight the tunability of the designed system, which can be applied when a “burst” release is preferred (continuous field), such as for treating a viral infection, or when a release needs to be repeated successively but dosing is challenging (pulsatile field), such as in the case of cytotoxic drugs in chemotherapy^35^,^36^,^37^,^38^.

#### Continuous release mode

**Figure 3:**
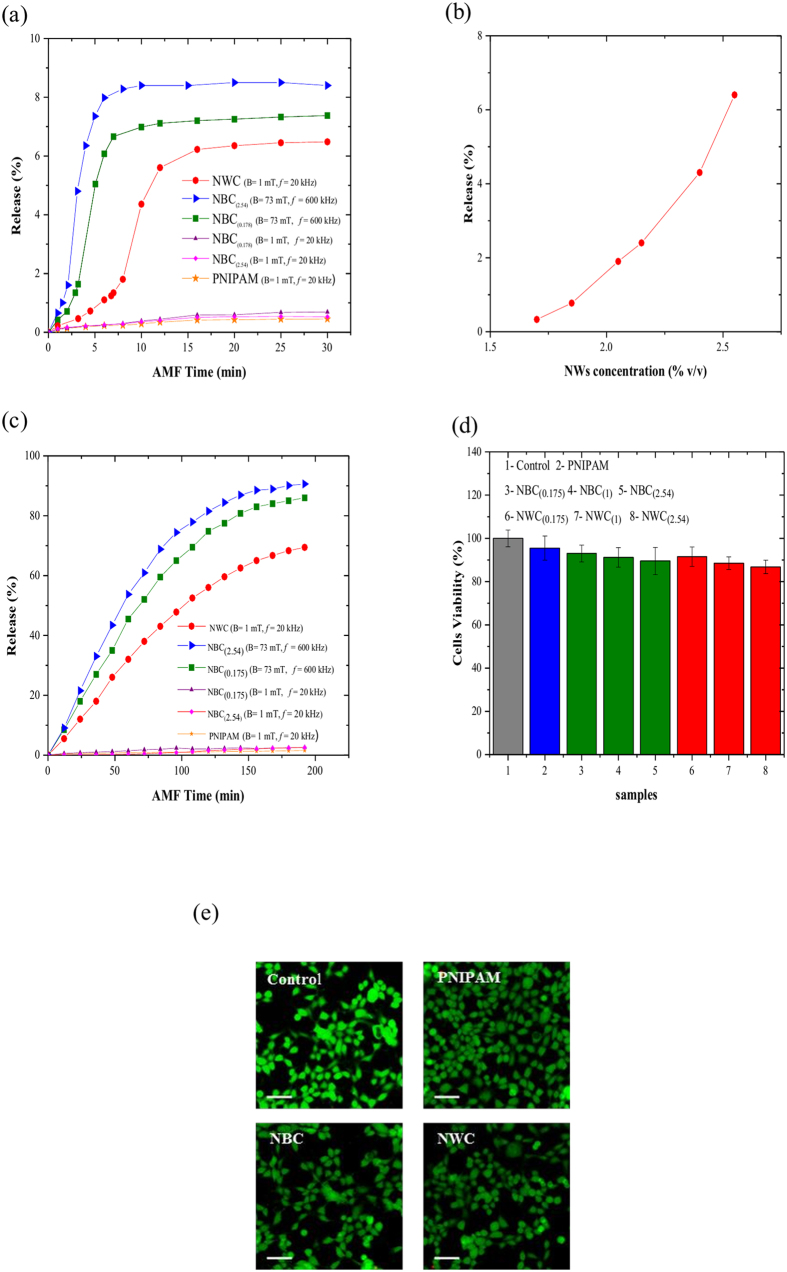
(**a**) Release of Rh(B) from particles at low-power and high-power alternating magnetic fields. At around 15 minutes, the release of NWC at low power filed was around 6.5% (**b**) Effect of NW concentration in NWC particles on the release of Rh(B). (**c**) Pulsatile release of Rh(B) from particles exposed to an alternating magnetic field for 12 minutes separated by 5 minute intervals. At around 200 minutes, NWC has around 70% release at low power field (**d,e**) Viability tests of HCT 116 cells after 24 hours of incubation with particles evaluated by MTT assay and calcein/EthD staining, respectively. The control experiment was done without any particles. Scale bar in (**e**) is 100 μm.

The temporal behavior of the release during the continuous mode is shown in (Fig. 3a). PNIPAM, NBC_(2.54)_ and NBC_(0.175)_ particles at low-power field release about 0.5% in 30 minutes. NWC particles at low-power field and NBC particles at high-power field have a much larger release with a profile that closely correlates with the shrinking and temperature change of the particles (Fig. 2b). After reaching the LCST the release increases sharply from approximately 2% to 6%, due to shrinking of the particles, acting like a hydrostatic pump that propels the dye outwards. The release saturates at about 6%, 7% and 8.5% in case of NWC, NBC_(0.175)_ and NBC_(0.254)_ particles, respectively. As expected, NBC_(2.54)_ has a higher release efficiency than NBC_(0.175)_ due to the higher power density, which results in a better swelling/deswelling behavior and hence a higher release rate^19^. It should be noted that the magnetic field was applied for over 100 minutes, but only a very small release of 0.3 to 0.5% per hour was observed, which is due to normal diffusion of Rh(B). The faster release process found for the NBC particles is in accordance with their faster responsive behavior at high-power AMF as shown in (Fig. 2b) and is related to the higher losses (see Supplementary material).

The effect of the concentration of NWs in the NWC particles on the release is shown in (Fig. 3b), when an AMF of B = 1 mT and f = 20 kHz was applied continuously for 15 minutes. Below 1.7% v/v of NWs, no significant release was observed, which is attributed to the fact that the heating produced by the NWs is not sufficient to generate a response. Between 1.7 and 2.54% v/v of NWs, the release increases rapidly from about 0.3% to about 6.5%. Within this concentration range, the temperature reaches the LCST, causing an increased release. We found that a further increase of the NW concentration adversely affects the polymerization of the gel and the integrity of the particles.

#### Pulsatile release mode

The pulsatile release mode was studied by applying AMF pulses of 12 minutes in duration, separated by 5 minute intervals. The pulse duration was chosen to obtain the maximum drug release from NWC particles as found for the continuous release mode (Fig. 3a). As shown in Fig. 3c, the NWC particles maintain a relatively constant release for each pulse, even after 16 cycles, and a total release of nearly 70% was achieved in less than 3.5 hours. Similar results were found for NBC particles using the high-power field. Unlike the continuous AMF mode in which the gel can collapse and possibly lead to a decrease of the pore dimensions or even clogging of the pores; the pulsatile mode enables the gel to re-swell, resulting in a re-opening of the pores for the next cycle^39^.

Figure 3c also shows that almost no release is obtained from PNIPAM and NBC particles in a pulsatile low-power AMF. The small release observed is due to Rh(B) diffusion.

### Cytotoxicity tests

Several studies have shown the biocompatibility of PNIPAM in gel form or with embedded iron or iron oxide NBs^40^,^41^. For PNIPAM with embedded NWs no data is available so far; hence, this study will elucidate whether the NWC particles are suitable for a wide range of *in vivo* and *in vitro* applications (hyperthermia, cancer therapy, gene release, tissue engineering,...). We evaluated the biocompatibility of the particles using an MTT assay on HCT 116 cells. Figure 3d shows the viability of cells incubated with PNIPAM, NBC and NWC particles, at different concentrations, for 24 hours. The average viability was around or above 90% compared to a control sample, implying that the NWC particles have the same biocompatibility with the model cell line as the NBC or the native PNIPAM particles.

Additionally, cells were stained with calcein AM/EthD-1 and then imaged using fluorescence microscopy to confirm the cytotoxicity results of the MTT assay. Calcein AM is a non-fluorescent, cell-permeant dye that emits an intense green fluorescence upon conversion to calcein by the intracellular esterases, enzymes that are only active in live cells. EthD-1, on the other hand, is impermeable to intact cell membranes. Dead cells have a compromised cell membrane, allowing the dye to enter and bind to the nucleic acids, emitting red fluorescence. The combination of the two dyes allows distinguishing between cells that are alive (stained green) from those that are dead or unhealthy (stained red or red/green). As can be observed in (Fig. 3e), the cell population shows a high cell esterase activity for PNIPAM, NBC and NWC particles.

In this work, we present a new kind of thermoresponsive particle, made of PNIPAM with embedded magnetic NWs. These nanocomposite particles, which were fabricated by a simple and fast microfluidic method, feature an extremely efficient heating mechanism, which enables remotely controlled release with a magnetic field of about five orders of magnitude less power compared to similar systems in which magnetic NBs as have been used. Such low-power magnetic fields could be realized on-chip, which would enable the integration of such particles in e.g. lab-on-chip systems or implantable sensors that do not require external field sources. Our results show that the NWC particles have a low cytotoxicity, making them relevant for *in-vivo* applications. The low-power magnetic field required to trigger NWC particles is harmless and can be generated by simple and compact equipment, which is an essential advantage for integrating and implanting devices in clinical and healthcare applications. The efficient heating mechanism provided by the magnetic NWs and utilized here to trigger a release from thermoresponsive particles could also be exploited for many other applications.

## Methods

Microfluidic chips for laminar flow or droplet based applications are usually fabricated using clean room technologies^42^,^43^,^44^,^45^,^46^,^47^. An alternative approach known as capillary microfluidic devices is adopted in this work^27^,^48^,^49^,^50^, where simple or multiple emulsions can be generated efficiently. As mentioned, the microfluidic devices (Figure S1e) were fabricated using microscope glass slides and glass capillaries and a flow focusing method was used to make the nanocomposite particles, generating an emulsion, in which two aqueous phases are mixed and encapsulated inside an oil stream. One of the aqueous phases contained either NWs or NBs. The NBs were commercially available iron oxide (20 nm, Micromod, Catalog No. 79-00-201). The NWs with 500 nm in length and 45 nm in diameter (Figure S1f) were fabricated via pulsed electrodeposition of iron in porous anodized aluminum oxide templates.

The thermoresponsive behavior of the particles was characterized by measuring the swelling/deswelling ratio:


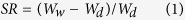


The experimental values of *Load*_*eff*_ were obtained by measuring the amount of Rh(B) by UV spectroscopy (Picrodrop 200, from Bioneer) from 10 mL of the water/Rh(B) solution, sampled before and after the loading procedure.

The low power magnetic field was generated with a Litz wire coil made of 60 strand wires (40 μm diameter per strands) with 6 layers and 8 turns per layer, which was connected to a power supply to generate a field of 1 mT amplitude and 20 kHz frequency. The high power magnetic stimuli was produced with an inductive heater (Induktive Erwärmungsanlagen GmbH, Austria, model No: TH3HT) that was configured to produce a magnetic field of 600 kHz and 73 mT.

For the AMF experiments, the particles were placed inside a plastic container at the center of the coil and the container was insulated by a Polydimethylsiloxane cover. For the release experiments, 100 mL of water with particles was placed in a flask. The amount of Rh(B) released from the particles at different times in the experiment was determined by sampling 10 mL from the flask before and after applying the AMF, using UV spectroscopy.

Cytotoxicity tests were conducted by means of an MTT (3-(4, 5-dimethylthiazol-2yl)-2, 5-diphenyl tetrazolium bromide) assay on HCT 116 colon carcinoma epithelial cells (ATCC-CCL274). Cell viability was also evaluated using a LIVE/DEAD Viability/Cytotoxicity Kit (Molecular Probes) in combination with a Leica DMI6000 B fluorescence microscope.

Further details on these experiments can be found in the Supplementary material.

## Additional Information

**How to cite this article**: Yassine, O. *et al*. Highly Efficient Thermoresponsive Nanocomposite for Controlled Release Applications. *Sci. Rep.* **6**, 28539; doi: 10.1038/srep28539 (2016).

## Supplementary Material

Supplementary Information

## Figures and Tables

**Figure 1:**
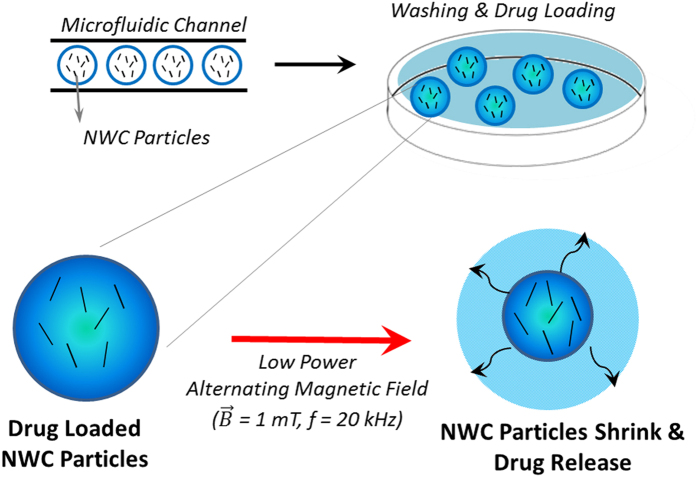
The nanowire composite (NWC) particles are fabricated in a microfluidic system and are loaded with a drug by means of diffusion. The heat generated by the nanowires upon the application of an alternating magnetic field of 1 mT in amplitude and 20 kHz in frequency causes the gel-structure to shrink in size, expelling the loaded drug.
