# Supplementary material for: Highly Efficient Thermoresponsive Nanocomposite for Controlled Release Applications
Source: Sci Rep. 2016 Jun 23;6:28539. doi: 10.1038/srep28539 (PMC4917869; doi:10.1038/srep28539)
Supplement: Supplementary Information [file srep28539-s1.doc]

**Supporting Information**

Highly Efficient Thermoresponsive Nanocomposite for Controlled Release Applications

**Omar Yassine1*+, Amir Zaher2+, Er Qiang Li3, Ahmed Alfadhel1, Jose Perez4, Mincho Kavaldzhiev1, Maria F. Contreras4, Sigurdur T. Thoroddsen3, Niveen M. Khashab4 and Jurgen Kosel1***

1Computer, Electrical and Mathematical Sciences & Engineering Division. King Abdullah University of Science and Technology (KAUST), Thuwal 23955-6900, Kingdom of Saudi Arabia

2School of Engineering. University of British Columbia, 3333 University Way. Kelowna, BC, V1V 1V7, Canada

3Physical Sciences and Engineering Division. King Abdullah University of Science and Technology, Thuwal 23955-6900, Kingdom of Saudi Arabia

4Biological and Environmental Sciences and Engineering Division. King Abdullah University of Science and Technology, Thuwal 23955-6900, Kingdom of Saudi Arabia

***Fabrication of Nanowire Composite Particles***

The microfluidic devices were designed and fabricated using microscope glass slides and glass capillaries 1,2. First, the glass slides and capillaries were cleaned in ultrasonic baths of acetone, isopropanol and ethanol for 10 minutes each. Then, two glass slides (75 × 25 mm2, thickness of 1 mm) were fixed onto a bigger microscope glass slide (75 × 50 mm2, 1 mm thick) using double-sided tape (Scotch permanent double sided tape). The glass capillaries of 1 mm in diameter were heated and pulled by a micro-capillary puller (P-1000, Sutter Instrument), to form tapered ends with the desired orifice sizes. A surface wettability modification of the capillaries and the channel was carried out by applying a commercial coating agent (FluoroPel PFC 801A, Cytonix Corporation or Glaco Mirror Coat ‘Zero’, Soft 99 Co.) as a water repellent. Next, two tapered capillaries were bonded inside of the channel with epoxy (HP 250, ITWDevcon, Inc.) with one of them just penetrating the opening of the other one. This both fixes the capillaries in place and separates the flows of the various liquids by forming an impenetrable barrier. Finally, a glass slide (75 × 25 mm2, 1 mm thick), serving as the cover of the microfluidic device, was bonded onto the channel using double-sided tape. Syringe needles were bonded to holes (1.7 mm in diameter) in the cover slide by epoxy, forming inlets. Diamond core drills (Eternal Tools) were used to fabricate these inlets.

To generate monodispersed ((W+W)/O) emulsions, liquid 1, liquid 2 and liquid 3 were used as the inner, middle and outer phases, respectively. Liquid 1 contained 20 % V/V of the monomer N-isopropylacrylamide (NIPAM), 2 mL of N,N,N’,N’-tetramethylenediamine (TEMED) as the accelerator for the reaction, 6 % V/V of the crosslinker, N,N-methylene(bis)acrylamide (BIS), and 0.5 mL [2-(methacryloyloxy) ethyltrimethyl ammonium chloride (METAC). Liquid 2 contained 4 % V/V ammonium persulfate (APS) and iron nanowires (NWs), or iron oxide nanobeads (NBs). Silicon oil was used as the oil phase. All chemicals were obtained from Sigma Aldrich.


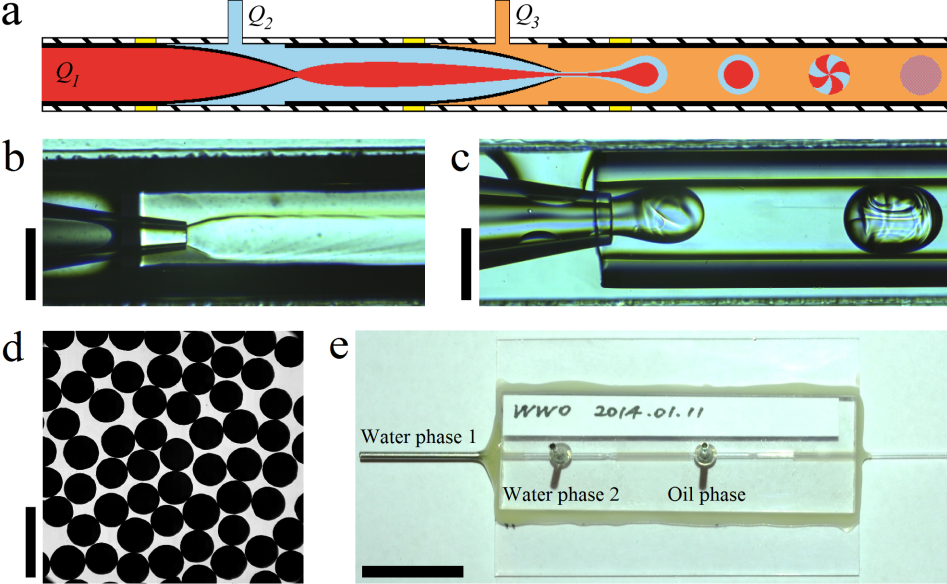

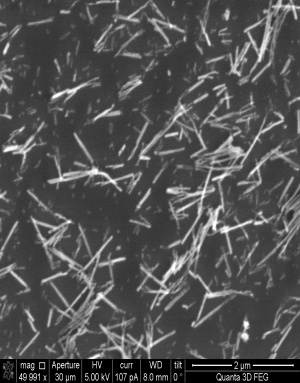


f

**Figure S1.** Fabrication of the NWC particles using the microfluidic approach (a) Schematic configuration of the microfluidic system for synthetizing the NWC particles. Q1 (20 µL min-1), Q2 (20 µL min-1) and Q3 (120 µL min-1) are the flow rates for the inner, middle and outer phases, respectively. (b) The two aqueous phases meet and form a continuous laminar flow at the outlet of the first capillary. (c) The generation of ((W+W)/O) emulsions at the outlet of the second capillary. (d) Magnified picture of magneto-thermoresponsive NWC particles fabricated with the capillary microfluidic device. (e) A typical microfluidic device fabricated for preparing the NWC particles. (f) SEM image of Fe NWs. Scale bars are 400 µm in (b) and c), 200 µm in (d) 2 cm in (e) and 2 µm in (f).

As shown in (**Figure S1a**), liquid 1 was fed into the channel through the first capillary, encountering liquid 2 at the outlet of the first capillary. Since the viscosities of liquid 1 and liquid 2 are close to each other, the local shear stress is not high enough to pinch-off liquid 1 into droplets, as shown in (**Figure S1b**). The microfluidic approach ensures laminar flows, preventing the two liquids from mixing in the second capillary, which would block it. When the two streams of liquid 1 and liquid 2 reach the outlet of the second capillary, the shear stress from liquid 3 pinches-off the streams into monodispersed droplets, as shown in (**Figure S1c**). Typical flow rates used are *Q*1 = 20 µL min-1 (liquid 1), *Q*2 = 2 µL min-1 (liquid 2) and *Q*3 = 120 µL min-1 (liquid 3). (**Figure S1d**) shows typical NWC particles fabricated with the capillary microfluidic device. The NWC particle size distribution can be described as PDI index, which is defined as
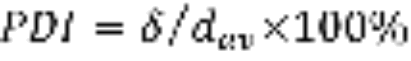
, where *δ* is the standard deviation and
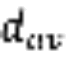
 is the average particle diameter. Images of more than 500 NWC particles are taken and analyzed by a Matlab program to extract the particle sizes. The calculated PDI value is 3.9%, indicating a highly monodisperse NWC particle generation with our device.

At the outlet of the device, the NWC particles were collected by a glass container filled with 100 mL of water. The NWC particles were washed with water and collected using a sieve. This washing procedure was repeated 3 times, followed by storage of the NWC particles in water at room temperature, ready for subsequent experiments.

***Fabrication of Iron Nanowires***

Iron NWs, 500 nm long and 45 nm in diameter (**Figure S1f)**, were produced via pulsed electrodeposition of iron in porous anodized aluminum oxide template membranes, as developed elsewhere 3-5.

High purity aluminum disc substrates (99.999%, Goodfellow) were rinsed and sonicated in acetone, then isopropanol, and finally distilled water, with the sequence repeated three times, and each solution sonication lasting 5 minutes. Next, discs were electropolished for 3 min at 25 V (2 A current limit), with the discs connected to the negative terminal and submerged in a stirred electropolishing solution (25% perchloric acid and 75% ethanol concentrations) at 4°C, along with a positive electrode (Pt mesh).

The polished substrates were then anodized in 0.3M oxalic acid at 2-4°C, applying a voltage of 40 V (positive electrode Pt mesh, negatively charged Al substrates, both in acid), for the first anodization step (24 hr). This generates slightly ordered nanopores whose ordering increases with depth of the grown anodized aluminum oxide layer. The aluminum oxide layer was dissolved at 37°C in 12 hr using a slow acting solution (1.8 g chromium VI oxide, 7.1 g phosphoric acid, 100 mL distilled water), leaving the bottom aluminum substrate with highly ordered indentations used as seeds for the second anodization process.

The second anodization (10 hr) used the same conditions as the first one and results in an approximately continuous level of ordering from top to bottom of the second anodized aluminum oxide layer. The resulting nanopores are approximately 45 nm in diameter and are hexagonally ordered.

An exponentially decaying voltage (40 V down to 4.5 V) and using oxalic acid at 2-4°C results in dendritic openings at the bottom of the pores, which allowed for electrical contact between the solution placed in the pores, and the substrate’s un-oxidized and conductive aluminum bottom (required for electrodeposition of metal).

Finally, pulsed electrodeposition (60 mA for 2 ms, 5 V for 2 ms, 1 s rest time) was used to deposit Fe NWs from a pH 4 electrodeposition solution (45 g/L of FeSO4·7H2O, 30 g/L of H3BO3, 1 g/L of C6H8O6 ascorbic acid). The NWs length was tailored using the deposition time. NWs were released from the aluminum oxide nanoporous membrane using fast acting 1M NaOH, and collected in plastic tubes (Eppendorf) using a magnetic rack (DynaMag-2, Invitrogen) for cleaning in ethanol and storage prior to use.

***Modeling of Nanowire Vibration in PNIPAM***

To explain the mechanism of NW heating, the power density (PD) required for triggering particle shrinkage is first determined by modeling NB power generation. The heat generated by the superparamagnetic NBs, when exposed to an alternating magnetic field, is a result of magnetic losses that occur due to two mechanisms: Neel relaxation and Brownian relaxation. Neel relaxation, *τN*, is associated with the reorientation of magnetic moments, whereas Brownian relaxation, *τB*, is associated with the reorientation of the entire particles. Neel relaxation can be expressed as 6:

$\tau_{N}=\tau_{o}\exp\left(\frac{KV_{m}}{k_{B}T}\right)$, (S1)

where *τ0* is the initial relaxation time, *K* is the magnetic anisotropy constant, *Vm* is the volume of the magnetic particle, *kB* is the Boltzmann constant and *T* is the temperature.

Brownian relaxation is expressed as:

$\tau_{B}=\frac{3\eta V_{H}}{k_{B}T}$, (S2)

where *η* is the medium viscosity, *VH* is the hydrodynamic volume of the magnetic particle. The viscosity of the environment in which a NB is suspended was estimated to be that of its surrounding aqueous solution contained in the hydrogel.

The volumetric power dissipated by NBs is proportional to the internal energy of the particles *∆U* and the frequency *f*, and can be expressed as 7:

$P=f\Delta U=\pi\mu_{0}\chi_{i}fH^{2}$, (S3)

where *H* is the magnetic field amplitude, *µ0* is the permeability of free space and *χi* is the imaginary part of the complex susceptibility, which depends on the frequency, Brownian and Neel relaxations. *χi* is dependent on the frequency, effective relaxation *τ*, and the static susceptibility *χ0=∂M/∂H* (which is obtained from the magnetization curves of the NBs):

$\chi_{i}=\frac{2\pi f\tau}{1+(2\pi f\tau)^{2}}\chi_{0}$, (S4)

where the effective relaxation time *τ* is calculated as:

$\frac{1}{\tau}=\frac{1}{\tau_{N}}+\frac{1}{\tau_{B}}$, (S5)

The volumetric power dissipation of NBCs with different NB volume ratio was calculated for. Using Equation S5, an alternating magnetic field of 73 mT in amplitude and 600 kHz in frequency, *K* = 13.5 kJ m-3, and *τ0* = 1 ns, the total power dissipated inside an NBC is PDnb = 5.8x10^5^ W/m3. PDnb is required for the microparticle to exhibit the experimentally observed shrinkage.

In order to study the heat generated by the vibrating NWs in the PNIPAM microparticles, the 2nd order non-linear ordinary differential equation obtained in previous studies 8-10 was adjusted for the case of a fixed direction AMF to determine the angle of rotation θl of one iron NW in PNIPAM during one magnetic field cycle:


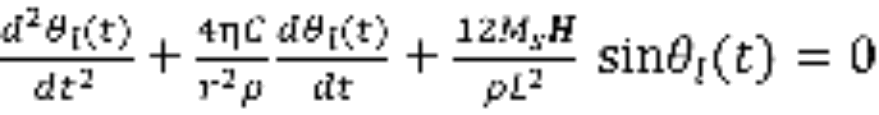
. (S6)

Here, θl(t) is defined as the angle between the nanowire and the fixed magnetic field direction with respect to time, C is a geometric factor, η is the viscosity, r is the radius of a the nanowire (22.5 nm approximately, measured via SEM imaging), ρ is the density of iron (7870 kg/m3), **H** is the sinusoidal AMF (1 mT), Ms is the magnetic saturation value for iron NWs (1.71x10^6^ A/m), and L is the length of the nanowire (500 nm). A value of the geometric constant was calculated, C = 0.1443, determined for a two segment NW with a 500 nm to 45 nm aspect ratio 9 (see following section for derivation of C). The viscosity of the environment in which a NW is suspended was estimated to be that of its surrounding aqueous solution contained in the hydrogel.

The total angle traveled by one nanowire in one full cycle, denoted by K (rad), is simply 2 times the absolute value difference between θl(0) at the beginning of a cycle, and θl(t/2) halfway through the cycle. The initial position of π/4 best represents the average NW position with respect to the fixed direction of the AMF, since each nanowire lies at an angle between 0 and π/2 with respect to the field, regardless of physical orientation or orientation of magnetization direction. The situation under a 20 kHz AMF with fixed direction is similar to the case discussed by Keshoju et al 9, in which the NW oscillates around its position, without fully rotating, while under a fast rotating magnetic field, with the difference being that the angle between the field direction and the NW start position in this study remains fixed.

The work in the first half of a cycle, W1 (N∙m), required to achieve an angular displacement K/2 for one NW, is found by integrating magnetic torque over the displaced angle:


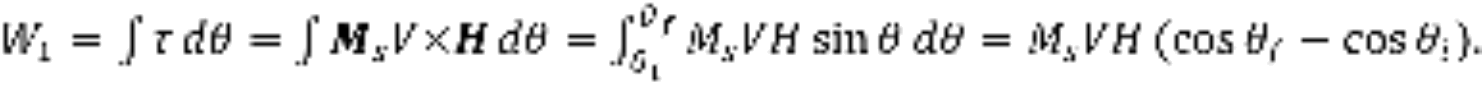
 (S7)

Here, Ms is the magnetization of the iron NW, H is the applied magnetic field strength, V is the volume of the NW, and MsV is the magnetic moment (Am2 or N∙m/T). It is found that K = 31.91º (K/2 = 15.96º per half cycle). The total work is the sum of W1, and W2 (the work done during the second half of the cycle), which are equal, therefore

Wtotal= 2∙MsVH∙cos(θf – θi). (S8)

Work input into the NWC microparticle is converted to heat generation; energy from the magnetic field converts to kinetic energy in each NW in the form of magnetic torque over angular displacement, which in turn converts to energy loss that is transferred from the NW to its surroundings via hydrodynamic friction.

Finally, the power generated by the nanowire, Pnw (W/s, or N∙m/s, per nanowire), was found by multiplying Wtotal with the magnetic field frequency (20 kHz), Pnw = Wtotal ∙ f. With a 2.54 % v/v concentration of Fe NWs, and a total volume of a particle Vparticle, the total number of nanowires Nnw in a particle is determined, Nnw = (0.0254Vparticle)/Vnw, and the total power of a NWC particle is then Pparticle = Pnw ∙ Nnw = 1.58E-6 W. The power density is then simply PDnw = Pparticle / Vparticle = 4.84x10^5^ W/m3, which matches well with the power density PDnb required for heating the NBC microparticles. The difference in PD values may account for the difference in shrinkage time in Fig. 2b. For model derivations, see the following section.

Similarly, for a NBC with 2.54 % v/v iron oxide content exposed to the lower power field (1mT, 20 kHz) the PDnb is found to be 1.76x10^-6^ W/m3, while that of a NWC with 2.54 % v/v iron NW exposed to the same low power field is PDnw = 4.84 x10^5^ W/m3, (2.75x1011 times larger). This explains why 2.54 % v/v iron oxide cannot generate enough heat to actuate the NBC while NWs can.

***Derivation of Equation of Motion for Vibrating Nanowires***

In the rotational motion of a NW, the torques counteracting each other are that of the fluid drag and that of the magnetic moment, expressed as τD and τM respectively:


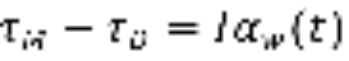
, (S9)


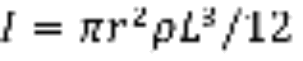
. (S10)

I is the moment of inertia of a wire, while ρ, r and L are its density, radius and length. The angular acceleration is αw (derivative of angular velocity ωw).

Below, Ms is the nanowire’s magnetization at saturation, H is the amplitude of the applied magnetic field, C is the geometric factor based on r, L, the dynamic viscosity of the fluid η, and the number of segments N used to describe the portions on a wire that are rotating around a fixed midpoint in our case (N=2). We use c1, c2 and D to represent constant factors in order to simplify the appearance of the equations (C, c­1, and c2­ represent unrelated terms). τD and τM in Eqn. (S9) are then expressed as:


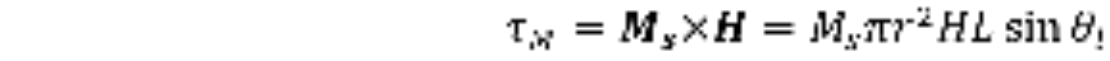
 (S11)

and


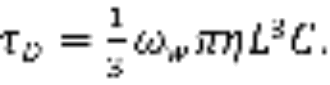
 (S12)

Here, the geometric factor C is
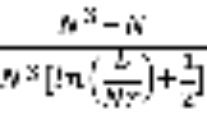
 . Using Eqn. S11 and Eqn. S12, Eqn. S8 becomes:
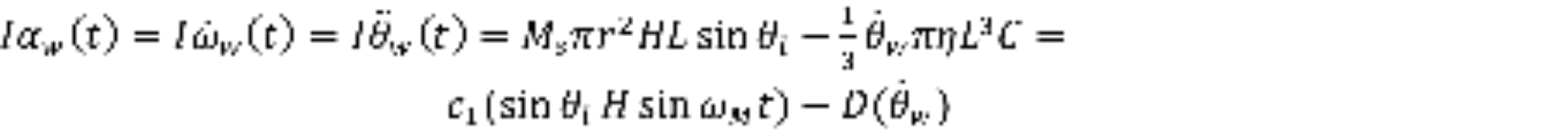
.

Rearranging this expression gives:


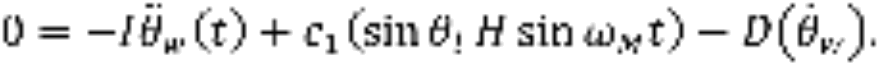
 (S13)

Here θl(t) is defined as the angle between the nanowire and the fixed magnetic field direction (i.e. the fixed line on which the field sinusoidally varies in polarity and magnitude following its own sinusoidal angular velocity ωM), θw(t) as the angle between the nanowire and its original position, and ωM is the magnetic field angular velocity. Knowing that
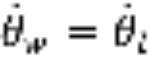
,
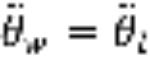
, and designating
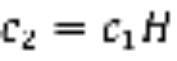
, then the following two terms are expressed:


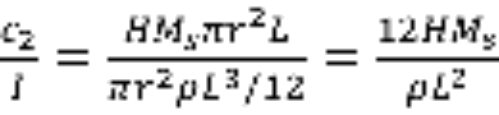
 , (S14)


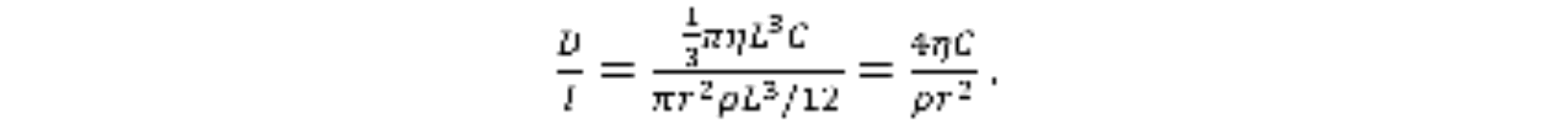
(S15)

Inserting these into Eqn. S13 gives
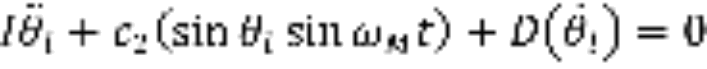
, re-written as:


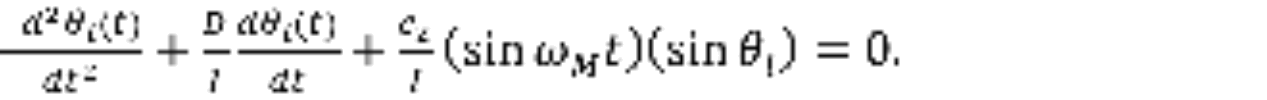
(S16)

In this form, equivalent to Eqn. S6, we can solve for θl(t).

***Cytotoxicity tests***

The MTT (3-(4, 5-dimethylthiazol-2yl)-2, 5-diphenyl tetrazolium bromide) assay was used to assess the cytotoxicity of theNWC. HCT 116 colon carcinoma epithelial cells (ATCC® CCL247TM) were cultured in McCoy’s 5A modified medium (Gibco®), supplemented with 10% fetal bovine serum (FBS) (Gibco®) and L-glutamine, and grown in a 37 ⁰C humidified incubator with 5% CO2. After reaching 80% confluence, cells were detached from the culture flasks with 0.25% trypsin-EDTA and counted using trypan blue staining. The Cells were cultured in 96-well plates and after 24 hours of stabilization they were treated with PNIPAM and NWC and NBC at different concentrations. After 24 hours incubation time, cell medium was discarded and replaced with 10% MTT solution —5 mg/mL in phosphate buffered saline (PBS)— in McCoy’s medium. The cells were incubated for two hours, and then the medium was discarded and replaced with 90% dimethyl sulfoxide (DMSO) – 10% sodium dodecyl sulfate lysis buffer to dissolve the MTT reduction products. The cell viability was evaluated through optical density (OD) with a microplate reader (XMarkTM, Bio-Rad) using a wavelength of 570 nm and a background wavelength of 630 nm.

Cell viability was also evaluated using a LIVE/DEAD Viability/Cytotoxicity Kit (Molecular ProbesTM). For this purpose, HCT 116 cells were cultured in 24-well plates and allowed to stabilize. The microparticles were then added to the culture. 24 hours later, the cell medium was discarded and the cells thoroughly washed with phosphate buffered saline (PBS). Then, 1.5 mL of 2 µM calcein AM and 4 µm EthD-1 working solution were added and the cells were incubated for 40 minutes at room temperature. The cells were washed with PBS and then imaged using a Leica DMI6000 B fluorescence microscope.

**References**

1 Li, E. Q., Zhang, J. M. & Thoroddsen, S. T. Simple and inexpensive microfluidic devices for the generation of monodisperse multiple emulsions. *Journal of Micromechanics and Microengineering* **24**, 015019 (015011 pp.)-015019 (015011 pp.) (2014).

2 Chu, L. Y., Kim, J. W., Shah, R. K. & Weitz, D. A. Monodisperse thermoresponsive microgels with tunable volume-phase transition kinetics. *Advanced Functional Materials* **17**, 3499-3504 (2007).

3 Vázquez, M., Pirota, K., Torrejón, J., Navas, D. & Hernández-Vélez, M. Magnetic behaviour of densely packed hexagonal arrays of Ni nanowires: Influence of geometric characteristics. *Journal of Magnetism and Magnetic Materials* **294**, 174-181 (2005).

4 Vázquez, M. *et al.* Magnetic properties of densely packed arrays of Ni nanowires as a function of their diameter and lattice parameter. *Journal of Applied Physics* **95**, 6642-6644 (2004).

5 Pirota, K., Navas, D., Hernández-Vélez, M., Nielsch, K. & Vázquez, M. Novel magnetic materials prepared by electrodeposition techniques: arrays of nanowires and multi-layered microwires. *Journal of alloys and compounds* **369**, 18-26 (2004).

6 Brown, W. F. Thermal fluctuations of a single-domain particle. *Physical Review* **130**, 1677-1682 (1963).

7 Rosensweig, R. E. Heating magnetic fluid with alternating magnetic field. *J. Magn. Magn. Mater.* **252**, 370-374 (2002).

8 Sun, L., Keshoju, K. & Xing, H. Magnetic field mediated nanowire alignment in liquids for nanocomposite synthesis. *Nanotechnology* **19**, 405603 (2008).

9 Keshoju, K., Xing, H. & Sun, L. Magnetic field driven nanowire rotation in suspension. *Applied Physics Letters* **91**, 123114 (2007).

10 Kim, K., Xu, X., Guo, J. & Fan, D. L. Ultrahigh-speed rotating nanoelectromechanical system devices assembled from nanoscale building blocks. *Nature communications* **5**, 3632 (2014).
